# CD44 Mediates Oral Squamous Cell Carcinoma-Promoting Activity of MRE11 via AKT Signaling

**DOI:** 10.3390/jpm12050841

**Published:** 2022-05-21

**Authors:** Shyng-Shiou F. Yuan, Amos C. Hung, Ching-Wei Hsu, Ting-Hsun Lan, Chang-Wei Su, Tsung-Chen Chi, Yu-Chiuan Chang, Yuk-Kwan Chen, Yen-Yun Wang

**Affiliations:** 1 Graduate Institute of Medicine, College of Medicine, Kaohsiung Medical University, Kaohsiung 807, Taiwan; yuanssf@kmu.edu.tw (S.-S.F.Y.); amos.hung@my.jcu.edu.au (A.C.H.); r070375@kmu.edu.tw (T.-C.C.); 2 Translational Research Center, Kaohsiung Medical University Hospital, Kaohsiung 807, Taiwan; 3 Department of Medical Research, Kaohsiung Medical University Hospital, Kaohsiung 807, Taiwan; 4 Center for Cancer Research, Kaohsiung Medical University, Kaohsiung 807, Taiwan; 980313@mail.kmuh.org.tw (C.-W.H.); 1060197@mail.kmuh.org.tw (C.-W.S.); yukkwa@kmu.edu.tw (Y.-K.C.); 5 Department of Obstetrics and Gynecology, Kaohsiung Medical University Hospital, Kaohsiung 807, Taiwan; 6 School of Dentistry, College of Dental Medicine, Kaohsiung Medical University, Kaohsiung 807, Taiwan; lanth@kmu.edu.tw; 7 Division of Oral and Maxillofacial Surgery, Department of Dentistry, Kaohsiung Medical University Hospital, Kaohsiung 807, Taiwan; 8 Division of Prosthodontics, Department of Dentistry, Kaohsiung Medical University Hospital, Kaohsiung 807, Taiwan; 9 Institute of Biomedical Sciences, National Sun Yat-Sen University, Kaohsiung 804, Taiwan; ycc@mail.nsysu.edu.tw; 10 Department of Dentistry, Kaohsiung Medical University Hospital, Kaohsiung 807, Taiwan; 11 Oral and Maxillofacial Imaging Center, Kaohsiung Medical University, Kaohsiung 807, Taiwan

**Keywords:** oral squamous cell carcinoma, CD44, cancer stemness, tumorsphere, metastasis, MRE11

## Abstract

Oral cancer is one of the highest-incidence malignancies worldwide, with the occurrence of oral squamous cell carcinoma (OSCC) being the most frequently diagnosed form. A barrier for oral cancer management may arise from tumor cells that possess properties of cancer stemness, which has been recognized as a crucial factor in tumor recurrence and metastasis. As such, understanding the molecular mechanisms underlying these tumor cells may provide insights for improving cancer treatment. MRE11 is the core protein of the RAD50/MRE11/NBS1 complex with a primary role in DNA damage repair, and it has been diversely associated with tumor development including OSCC. In this study, we aimed to investigate the engagement of CD44, a cancer stemness marker functioning in the control of cell growth and motility, in OSCC malignancy under the influence of MRE11. We found that overexpression of MRE11 enhanced CD44 expression and tumorsphere formation in OSCC cells, whereas knockdown of MRE11 reduced these phenomena. In addition, the MRE11-promoted tumorsphere formation or cell migration ability was compromised in OSCC cells carrying siRNA that targets CD44, as was the MRE11-promoted AKT phosphorylation. These were further supported by analyzing clinical samples, where higher CD44 expression was associated with lymph node metastasis. Additionally, a positive correlation between the expression of MRE11 and CD44, or that of CD44 and phosphorylated AKT, was observed in OSCC tumor tissues. Finally, the expression of CD44 was found to be higher in the metastatic lung nodules from mice receiving tail vein-injection with MRE11-overexpressing OSCC cells compared with control mice, and a positive correlation between CD44 and phosphorylated AKT was also observed in these metastatic lung nodules. Altogether, our current study revealed a previously unidentified mechanism linking CD44 and AKT in MRE11-promoted OSCC malignancy, which may shed light to the development of novel therapeutic strategies in consideration of this new pathway in OSCC.

## 1. Introduction

Oral cancer is a significant health issue worldwide with the notion that oral squamous cell carcinoma (OSCC) constitutes the majority of oral cancer incidence [1,2]. The management of OSCC remains challenging owing to the frequent occurrence of treatment resistance and metastasis [3,4,5]. These hurdles may arise from the complexity of the oral tumor microenvironment, where the heterogeneity of tumor composition plays a key role in control of tumorigenesis, malignant progression, and treatment response, but the underlying mechanisms are yet to be fully elucidated [6,7]. One mechanism for the recurrence or metastasis in OSCC has been proposed through alteration of cancer stemness, by which the cancer stem-cell-like properties, such as self-proliferation and migration ability in OSCC cells, can be augmented in adaptation to environmental stresses, including those resulting from chemotherapy and radiation therapy [8,9,10].

CD44 is a cell surface transmembrane glycoprotein belonging to the family of cell adhesion molecules with a broad function regarding cell growth and motility [11,12]. Of particular note, CD44 is also identified as a cancer stemness marker in a number of cancer types including OSCC, and the expression level of CD44 has been found to be positively associated with tumor development and progression [13,14,15]. Through binding interactions of CD44 with its natural ligands, such as hyaluronic acid or other binding partners secreted in the extracellular matrix, a series of signaling cascades in OSCC cells may be activated, leading to the enhanced tumor growth, metastatic ability, and resistance to cancer treatment [16]. Therefore, targeting CD44 and its signaling pathways that involve in the cancer-promoting activities has emerged as a novel therapeutic potential for OSCC [17,18].

Most conventional cancer treatments are known to induce DNA damages that cause cell death through the delivery of chemotherapeutic agents or ionizing radiation to the tumor sites [19]. However, some tumor cells possessing properties of cancer stemness may develop into a treatment-resistant cell population via potentiation of their DNA damage repair abilities [20]. As such, the differential expression of MRE11, a critical DNA damage response protein forming the core of the MRE11/RAD50/NBS1 complex for the repair of DNA double-strand breaks [21], has been associated with various malignancies [22,23]. Our group recently reported that elevated MRE11 expression is associated with increased OSCC tumor growth and metastasis; in addition, a higher expression level of MRE11 in patients is predictive of poorer survival under radiation therapy compared to those with a lower expression level of MRE11 [24]. This raised a hypothesis that the oncogenic effect of MRE11 on OSCC may involve the enhancement of cancer stemness. Therefore, the present study aimed to investigate this possibility by analyzing the effect of MRE11 on CD44 expression in OSCC cells, as well as the role of CD44 in MRE11-promoted OSCC malignancy. The clinical relevance of CD44 expression in OSCC patients was also investigated. Furthermore, the involvement of AKT, a potential signaling molecule associated with the CD44 pathway [14], was explored under this paradigm.

## 2. Materials and Methods

### 2.1. Patient Specimens

Oral tumor tissues were obtained from patients who were diagnosed with OSCC and underwent surgical treatment at the department of Oral and Maxillofacial Surgery, Kaohsiung Medical University Hospital, Taiwan, during the period between January 1999 and December 2014. Patients without a history of oral malignancies were included in the current study, while patients who were beyond the age of 18–80 years old were excluded from the current study (*n* = 88). The tumor grading was classified according to the American Joint Committee on Cancer (AJCC) Cancer Staging Manual (7th edition), and lymph node metastasis was determined by examining the presence of invading tumors in the regional lymph nodes. The current study involving human subjects was approved by the Institutional Review Board of Kaohsiung Medical University Hospital (KMUH-IRB-20130300), and written informed consents were obtained from all participants.

### 2.2. Cell Culture

Human oral squamous cancer cell lines CAL-27, HSC-3, and Ca9-22 were obtained from the Bioresource Collection and Research Center of Taiwan (https://www.bcrc.firdi.org.tw/ (accessed on 10 April 2022)), and cultured in Dulbecco’s Modified Eagle Medium (DMEM; Thermo Fisher Scientific, Waltham, MA, USA) supplemented with 10% fetal bovine serum (FBS; Biological Industries, Beit Haemek, Israel) and antibiotics (100 units/mL penicillin, 100 μg/mL streptomycin, and 2.5 μg/mL amphotericin B; Biological Industries, Beit Haemek, Israel). All cells were maintained at 37 °C in a humidified 5% CO_2_ incubator.

### 2.3. Gene Overexpression and Knockdown

Overexpression of MRE11 was carried out by infection of OSCC cells with pre-packaged lentiviral particles carrying pReceiver-Lv105 vector which expresses full-length human MRE11 gene (Accession no. BC005241.1) or empty pReceiver-Lv105 vector as control (GeneCopoeia). Knockdown of MRE11 was carried out by infection of OSCC cells with pre-packaged lentiviral particles carrying pLKO.1-puro vector which expresses short hairpin RNA (shRNA) targeting human MRE11 (5′-GCTGCGTATTAAAGGGAGGAA-3′) or firefly luciferase as control (National RNAi Core Facility, Academia Sinica, Taiwan). The designated viral solutions were added to cells in the cell culture medium containing 8 μg/mL polybrene (Merck, Darmstadt, Germany). After infection for 48 h, 2 μg/mL puromycin was added to the cell culture medium for selection. Surviving cells were subsequently maintained in the presence of 2 μg/mL puromycin until further experiments.

Knockdown of CD44 was carried out by transfection of OSCC cells with the small interfering RNA (siRNA; 5′-UAUUCCACGUGGAGAAAAATT-3′) targeting human CD44 or with the siRNA containing a scramble sequence as control (Thermo Fisher Scientific, Waltham, MA, USA). The cells were added to a mixture of designated siRNA and Lipofectamine 2000 (Thermo Fisher Scientific, Waltham, MA, USA) dissolved in Opti-MEM medium (Thermo Fisher Scientific, Waltham, MA, USA) for 6 h, followed by replacement with complete cell culture medium for 24 h before further experiments.

### 2.4. Western Blot

Total protein lysates of OSCC cells were extracted with RIPA buffer (Merck, Germany), and equal amounts of the extracted proteins from each sample were loaded for sodium dodecyl sulphate-polyacrylamide gel electrophoresis (SDS-PAGE) with the Mini-PROTEAN electrophoresis system (BIO-RAD, Hercules, CA, USA), followed by transferring the proteins onto PVDF membranes (Merck, Darmstadt, Germany) with the Mini Trans-Blot system (BIO-RAD, Hercules, CA, USA). The membranes were blocked with 5% non-fat milk for 1 h at room temperature and then incubated with designated primary antibodies at 4 °C overnight. After washing with TBST (Tris-buffered saline containing 0.1% Tween-20; Merck, Darmstadt, Germany), the membranes were incubated with species-matched horseradish peroxidase-conjugated secondary antibodies (Thermo Fisher Scientific, Waltham, MA, USA) for 1 h at room temperature. Immunoreactive proteins were detected by immersing the membranes with Immobilon Western chemiluminescent reagents (Merck, Darmstadt, Germany), and the images were acquired by ChemiDoc XRS+ imaging system (BIO-RAD, Hercules, CA, USA). Quantitation for protein expression was performed by Image Lab software (BIO-RAD, Hercules, CA, USA). The primary antibodies used for Western blot in this study are listed as follows: goat anti-MRE11 polyclonal antibody (Santa Cruz Biotechnology, Dallas, TX, USA); rabbit anti-CD44 polyclonal antibody (GeneTex, Hsinchu, Taiwan); rabbit anti-phospho-AKT (Ser473) polyclonal antibody (Cell Signaling Technology, Danvers, MA, USA); rabbit anti-AKT monoclonal antibody (clone C67E7; Cell Signaling Technology, Danvers, MA, USA); mouse anti-β-actin monoclonal antibody (clone AC-15; Merck, Darmstadt, Germany); rabbit anti-α-tubulin polyclonal antibody (GeneTex, Hsinchu, Taiwan).

### 2.5. Tumorsphere Formation Assay

The tumorsphere formation assay was carried out based on an established method [25] and our previous reports [26,27]. OSCC cells were re-suspended in phenol red-free DMEM (Thermo Fisher Scientific, Waltham, MA, USA) containing 20 ng/mL recombinant human epidermal growth factor (Peprotech, Rehovot, Israel), 20 ng/mL recombinant human fibroblast growth factor-basic (Peprotech, Rehovot, Israel), 1 × B27 (Thermo Fisher Scientific, Waltham, MA, USA), and 10 μg/mL insulin (Thermo Fisher Scientific, Waltham, MA, USA), followed by plating these cells in ultra-low attachment 96-well plates (5 × 10^2^ cells/well; Corning, New York, NY, USA) and culturing for 14 days. Images of tumorspheres were captured by the Eclipse Ti-S microscope (Nikon, Tokyo, Japan), and the formation of tumorspheres with diameter > 50 μm in each well was analyzed by ImageJ software (https://imagej.nih.gov/ij/ (accessed on 10 April 2022)).

### 2.6. Transwell Cell Migration Assay

OSCC cells were re-suspended in serum-free DMEM and plated in the inserts (2 × 10^4^ cells/insert; 8 μm pore size) of the 24-well transwell system (Corning, New York, NY, USA). The bottom wells were supplied with complete cell culture medium. After 24 h under normal cell culture conditions, cells remaining on the upper side of the insert membrane were removed by cotton swabs, while cells migrating to the underside of the insert membrane were fixed with 4% formaldehyde (Merck, Darmstadt, Germany) and stained with 0.05% crystal violet (Merck, Darmstadt, Germany). Images of the crystal violet staining were captured by the Eclipse Ti-S microscope and analyzed by ImageJ software.

### 2.7. Immunohistochemistry (IHC)

Formalin-fixed, paraffin-embedded tumor tissue sections were immunostained using the Bond-Max automated IHC stainer (Leica Microsystems, Wetzlar, Germany) according to the manufacturer’s instructions and our previous report [24]. The primary antibodies used for IHC staining in this study are listed as follows: goat anti-MRE11 polyclonal antibody (Santa Cruz Biotechnology, Dallas, TX, USA); rabbit anti-CD44 polyclonal antibody (GeneTex, Hsinchu, Taiwan); rabbit anti-phospho-AKT (Ser473) monoclonal antibody (clone 736E11; Cell Signaling Technology, Danvers, MA, USA). The percentage of positively stained tumor cells in the tissue sections was scored as 0 (0–4%), 1 (5–24%), 2 (25–49%), 3 (50–74%), or 4 (75–100%), and the intensity of staining was scored as 0 (negative), 1 (weak), 2 (moderate), or 3 (strong). The total score for each tissue section was calculated through multiplying the score of the percentage of positively stained cells by the score of the intensity of staining. For analysis of the association between CD44 expression and clinicopathologic characteristics, total scores ≤ 9 were categorized as low CD44 expression, and those > 9 were categorized as high CD44 expression. Images of IHC were captured by the Eclipse E600 microscope (Nikon, Tokyo, Japan), and scoring of the immunostaining was determined blindly and independently for each specimen by two pathologists under the same imaging conditions.

### 2.8. Metastatic Mouse Model

The animal study was approved by the Institutional Animal Care and Use Committee, Kaohsiung Medical University (IACUC approval no. 106094), and conformed to the ARRIVE guidelines 2.0 for preclinical animal studies. The procedures were adopted from our previous reports [24,28]. MRE11-overexpressing CAL-27 cells, or empty vector-expressing CAL-27 cells as control, were re-suspended in phosphate-buffered saline (2.5 × 10^5^ cells per mouse) followed by intravenous injection through the tail vein in six-week-old male non-obese diabetic/severe combined immunodeficient (NOD/SCID) mice (n = 7 per group) obtained from the National Laboratory Animal Center of Taiwan (https://www.nlac.narl.org.tw/ (accessed on 10 April 2022)). The mice were housed in specific pathogen-free grade cages at the Center for Laboratory Animals, Kaohsiung Medical University. Eight weeks post-injection, the mice were euthanized and sacrificed, and the lungs were collected for further analysis by immunohistochemistry.

### 2.9. Oncomine Database Analysis

CD44 mRNA expression levels in OSCC tissues and non-cancerous oral tissues were analyzed using the Oncomine database (previously operated on https://www.oncomine.org/ (accessed on 24 July 2021)), which was established for comparisons of differential gene expression [29]. All data analyzed on the Oncomine platform were log-transformed and illustrated by median-centered boxplots as default.

### 2.10. The Cancer Genome Atlas (TCGA) Database Analysis

CD44 RNA-Seq datasets of the head and neck squamous cell carcinoma (HNSC) in TCGA database were retrieved from TCGA website (Project ID: TCGA-HNSC; https://portal.gdc.cancer.gov/ (accessed on 5 August 2021)). Kaplan–Meier curves were applied to compare the overall survival probability between low expression and high expression of CD44 cohorts from the TCGA-HNSC patients.

### 2.11. Statistical Analysis

Statistical analysis was performed using SPSS software (IBM, Armonk, NY, USA). Chi-square test was used to assess the association between CD44 expression and clinicopathologic characteristics. Log-rank test was used for overall survival analysis via TCGA database. Student’s *t*-test was used for comparisons between experimental and control groups. Pearson correlation was used to evaluate the correlation of protein expression by IHC. For in vitro studies, data were presented as mean ± SEM from three independent experiments. The results were considered statistically significant if *p* < 0.05.

## 3. Results

### 3.1. Expression of MRE11 and CD44 in OSCC Patients

**Figure 1:**
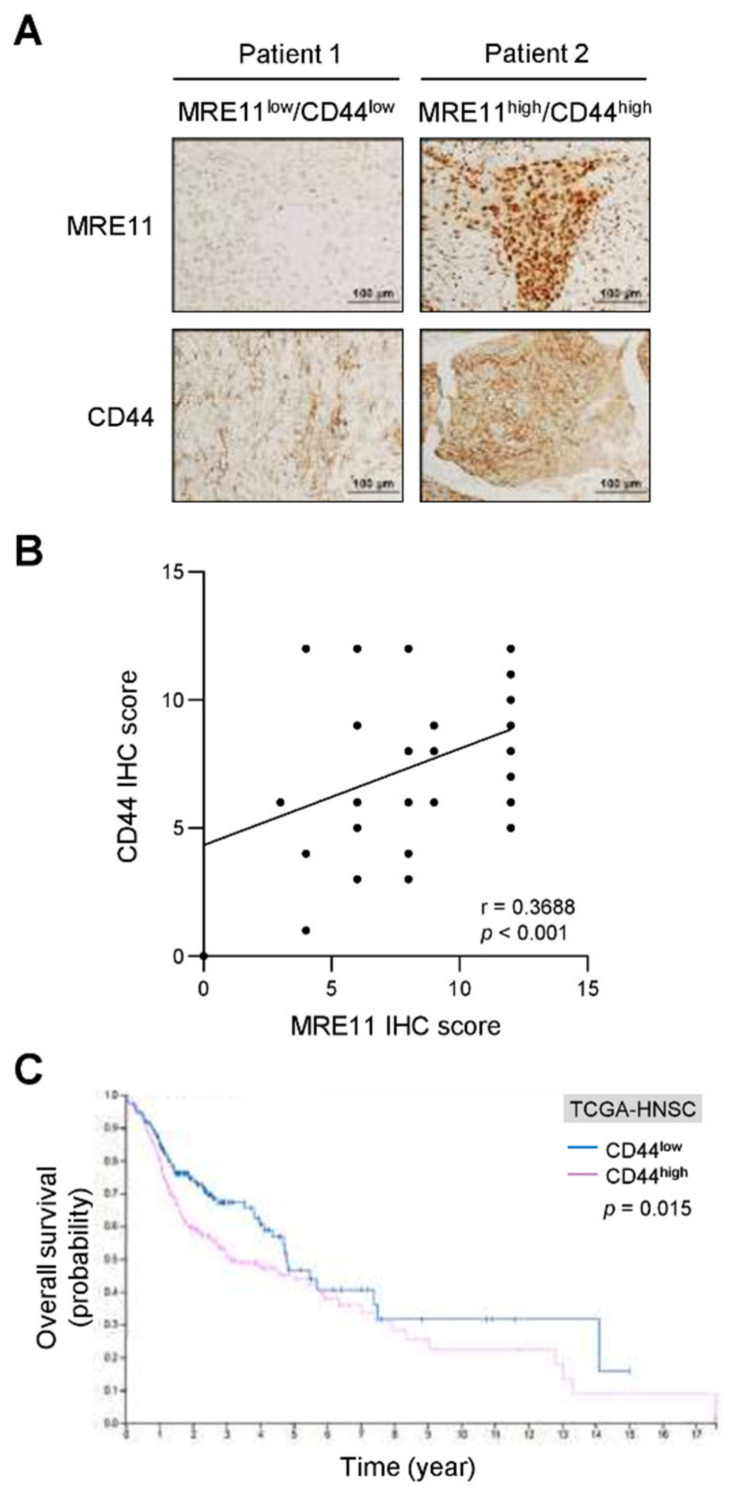
Correlation analysis for the expression of MRE11 and CD44 in OSCC tumor specimens and survival analysis for differential CD44 expression levels in OSCC patients. (**A**,**B**) Representative images of immunohistochemistry (IHC) staining for MRE11 and CD44 expression in the tumor tissue sections from OSCC patients were presented in (**A**). The quantitative IHC scores were analyzed with Pearson correlation (r) between MRE11 and CD44 in (**B**) (*n* = 88). (**C**) The overall survival of OSCC patients was analyzed via TCGA RNA-Seq datasets (TCGA-HNSC) for the low CD44 expression group (CD44^low^; *n* = 192) versus the high CD44 expression group (CD44^high^; *n* = 307) with log-rank test.

To evaluate the expression level of MRE11 and CD44 in OSCC patients, we analyzed the two proteins by immunohistochemical staining, which showed that a positive correlation between MRE11 and CD44 protein expression was observed in OSCC tumor tissues (*p* < 0.001; Figure 1A,B). We also analyzed CD44 expression in OSCC patients via the Oncomine database, revealing that the CD44 expression level was higher in OSCC tumor tissues compared to non-cancerous controls (*p* = 0.002; Supplementary Figure S1). Notably, the elevated CD44 expression was found to be associated with lymph node metastasis in our OSCC cohorts (*p* = 0.040; Table 1). In addition, a worse overall survival rate was observed in patients with higher CD44 expression compared to those with lower CD44 expression in the TCGA-HNSC cohorts (*p* = 0.015; Figure 1C). These findings suggest that the elevated CD44 expression in OSCC tumor tissues may serve a purpose for diagnosis and prognosis as proposed [30]. Moreover, these clinical observations suggest a potential for MRE11 that may influence CD44 expression in the regulation of cell behaviors of OSCC.

### 3.2. MRE11 Regulates CD44 Expression and Tumorsphere Formation in OSCC Cells

**Figure 2:**
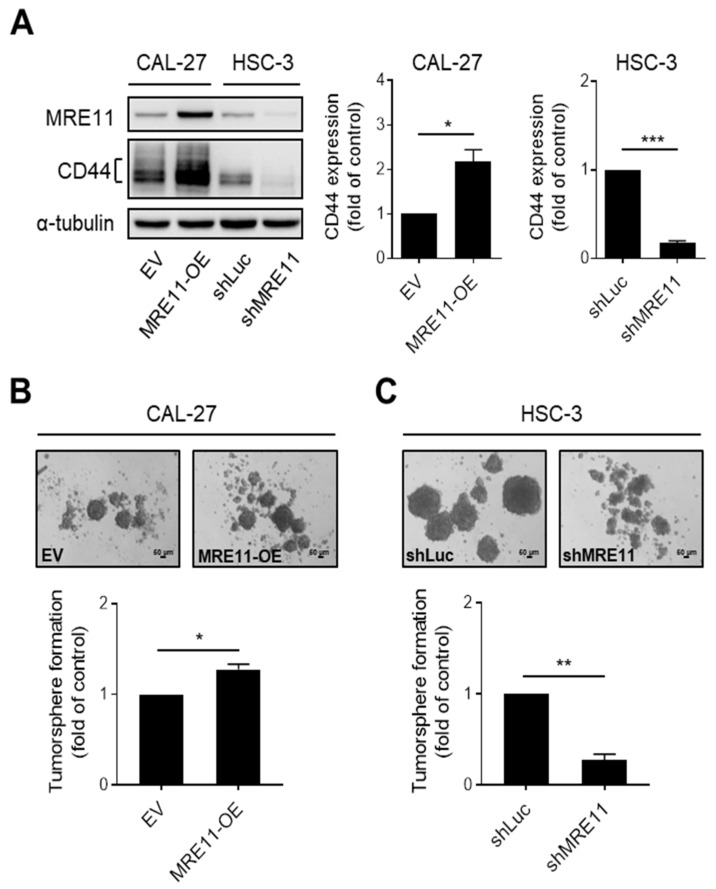
MRE11 regulates CD44 expression and tumorsphere formation in OSCC cells. (**A**) Total protein lysates from CAL-27 cells carrying overexpression vector of MRE11 (MRE11-OE) or empty vector (EV) and from HSC-3 cells carrying shRNA knockdown vector targeting MRE11 (shMRE11) or shRNA vector targeting firefly luciferase (shLuc) were collected and analyzed by Western blot. (**B**,**C**) CAL-27 cells carrying overexpression vector of MRE11 (MRE11-OE) or empty vector (EV) in (**B**) and HSC-3 cells carrying shRNA knockdown vector targeting MRE11 (shMRE11) or shRNA vector targeting firefly luciferase (shLuc) in (**C**) were collected and re-plated in ultra-low attachment microplates for tumorsphere formation assay. Quantitation of tumorspheres was carried out for those with diameter > 50 μm. Data were obtained from three independent experiments and presented as mean ± SEM. Statistical difference was determined by Student’s *t*-test. * *p* < 0.05, ** *p* < 0.01, *** *p* < 0.001.

To explore the potential that MRE11 that may affect CD44 expression, we established MRE11 overexpression or knockdown models in OSCC cells for the following investigations. First, we found that overexpression of MRE11 resulted in an increased CD44 protein expression in CAL-27 (Figure 2A) and Ca9-22 OSCC cells (Supplementary Figure S2A), whereas knockdown of MRE11 reduced the protein expression of CD44 in HSC-3 OSCC cells (Figure 2A), suggesting that CD44 expression is regulated downstream of MRE11. As CD44 is a hallmark for cancer stemness in a number of cancers, including OSCC [9,31], we further examined the ability of cancer stem cell-like growth in OSCC cells under the influence of differential MRE11 expression levels. As shown in Figure 2B and Supplementary Figure S2B, the ability of tumorsphere formation, which represents cancer cell growth from self-proliferation [32], was enhanced in MRE11-overexpressing CAL-27 and Ca9-22 cells, respectively. On the contrary, knockdown of MRE11 in HSC-3 cells showed a reduced capacity of tumorsphere formation (Figure 2C). These results suggest that MRE11 may promote malignant behaviors of OSCC by enhancing cancer stemness properties, such as increased CD44 expression and tumorsphere growth ability in OSCC cells.

### 3.3. CD44 Mediates MRE11-Promoted AKT Phosphorylation, Tumorsphere Formation, and Migration Ability in OSCC Cells

**Figure 3:**
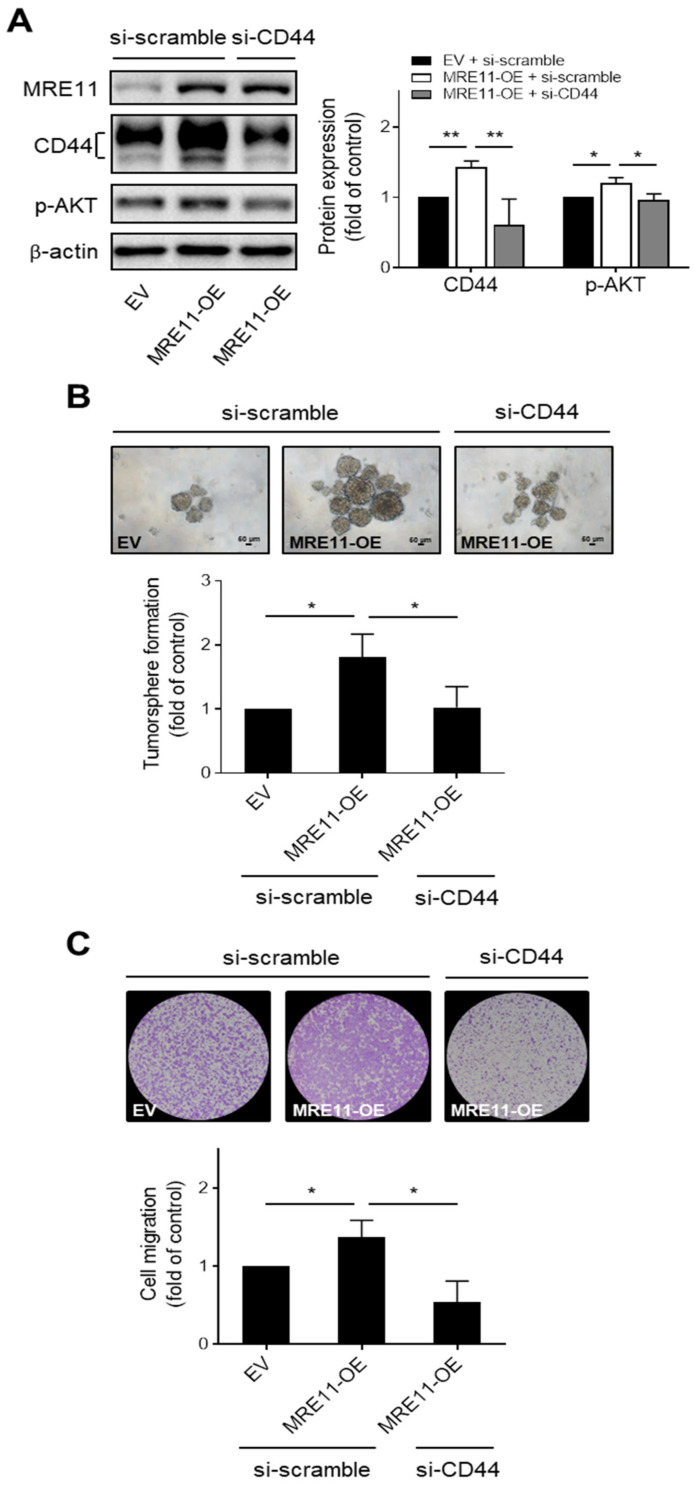
CD44 mediates MRE11-promoted AKT phosphorylation, tumorsphere formation, and cell migration in OSCC cells. (**A**) Total protein lysates from CAL-27 cells carrying (i) empty vector (EV) and siRNA containing a scramble sequence (si-scramble), (ii) overexpression vector of MRE11 (MRE11-OE) and siRNA containing a scramble sequence (si-scramble), or (iii) overexpression vector of MRE11 (MRE11-OE) and siRNA targeting CD44 (si-CD44), were collected and analyzed by Western blot. (**B**,**C**) CAL-27 cells carrying (i) empty vector (EV) and siRNA containing a scramble sequence (si-scramble), (ii) overexpression vector of MRE11 (MRE11-OE) and siRNA containing a scramble sequence (si-scramble), or (iii) overexpression vector of MRE11 (MRE11-OE) and siRNA targeting CD44 (si-CD44), were collected and re-plated in ultra-low attachment microplates for tumorsphere formation assay in (**B**), or re-plated in the inserts of transwell plates for transwell cell migration assay in (**C**). Quantitation of tumorspheres was carried out for those with diameter > 50 μm. Data were obtained from three independent experiments and presented as mean ± SEM. Statistical difference was determined by Student’s *t*-test. * *p* < 0.05, ** *p* < 0.01.

The role of CD44 in MRE11-promoted malignant behaviors of OSCC cells was further investigated in the MRE11 overexpression model combined with gene silencing for CD44. We found that the phosphorylation of AKT, a downstream effector of the CD44 pathway identified in several types of cancer cells [33,34,35], were elevated in MRE11-overexpressing CAL-27 and Ca9-22 cells transfected with scramble siRNA, whereas these phenomena were reversed in the counterpart MRE11-overexpressing OSCC cells transfected with siRNA that targets CD44 (Figure 3A and Supplementary Figure S3A, respectively). Additionally, the increased tumorsphere formation in MRE11-overexpressing CAL-27 and Ca9-22 cells was suppressed when these cells were transfected with siRNA targeting CD44 compared to those transfected with scramble siRNA (Figure 3B and Supplementary Figure S3B, respectively). Our previous report showed that overexpression of MRE11 promoted the migration ability of OSCC cells [24]. As the ability of cell migration has been recognized as an important trait of cancer stemness for the potential of dissemination of cancer cells from one tumor niche to another [36,37], we further examined whether CD44 involves in this MRE11-promoted activity. Our results showed that the enhanced ability of cell migration in MRE11-overexpressing CAL-27 and Ca9-22 cells was reversed in these cells that were transfected with siRNA targeting CD44 compared to those transfected with scramble siRNA (Figure 3C and Supplementary Figure S3C). These data together suggest that CD44 may crucially mediate MRE11-promoted malignant behaviors of OSCC cells, such as enhanced tumorsphere growth and cell migration ability, through activation of AKT signaling.

### 3.4. Correlation between CD44 Expression and AKT Phosphorylation in Tumor Tissues from OSCC Patients and In Vivo Metastatic Xenograft Mice

**Figure 4:**
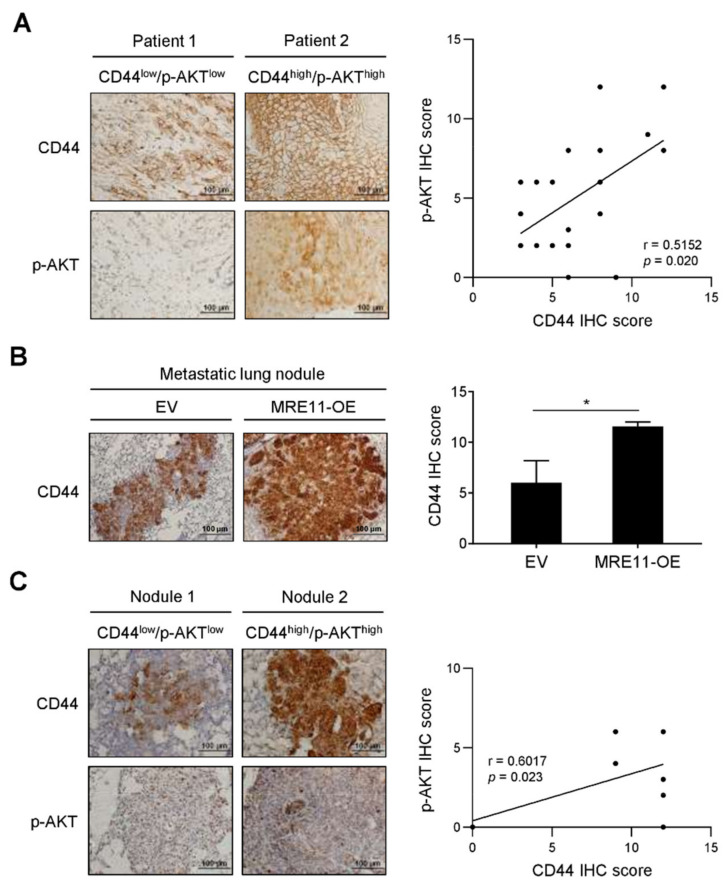
Correlation analysis for the expression of CD44 and AKT phosphorylation in OSCC patients and metastatic mouse model. (**A**) Representative images were presented for immunohistochemistry (IHC) staining of CD44 and phosphorylated AKT (p-AKT) expression in the tumor tissue sections from OSCC patients. The quantitative IHC scores were analyzed by Pearson correlation (r) between CD44 and p-AKT (*n* = 20). (**B**,**C**) CAL-27 cells carrying overexpression vector of MRE11 (MRE11-OE) or empty vector (EV) were collected and injected intravenously through the tail vein in mice. The lungs were collected after 8 weeks post-injection for IHC analysis. In (**B**), representative images were presented for IHC staining of CD44 in the metastatic lung nodules, and the quantitative IHC scores were presented as mean ± SEM (*n* = 7 per group). Statistical difference was determined by Student’s *t*-test. * *p* < 0.05. In (**C**), representative images were presented for IHC staining of CD44 and p-AKT expression in the metastatic lung nodules from xenograft mice. The quantitative IHC scores were analyzed by Pearson correlation (r) between CD44 and p-AKT (*n* = 14).

The expression of CD44 and AKT phosphorylation were further investigated for their clinical and in vivo relevance in relation to our in vitro findings. Notably, the expression of CD44 was found to positively correlate with the expression of phosphorylated AKT in the tumor tissues of OSCC patients (*p* = 0.020; Figure 4A). The in vivo metastasis with regard to different MRE11 expression levels of OSCC cells was evaluated in a tail vein-injected xenograft mouse model. As shown in Figure 4B, the mice injected with MRE11-overexpressing CAL-27 cells developed metastatic lung nodules that expressed a higher level of CD44 compared to that expression in control mice. Correlation analysis for these metastatic lung nodules revealed a positive correlation between the expression of CD44 and phosphorylated AKT (*p* = 0.023; Figure 4C). These results further support the findings from our in vitro investigation, and collectively suggest that the CD44-associated AKT pathway may crucially mediate the MRE11-promoted OSCC malignancy.

## 4. Discussion

**Figure 5:**
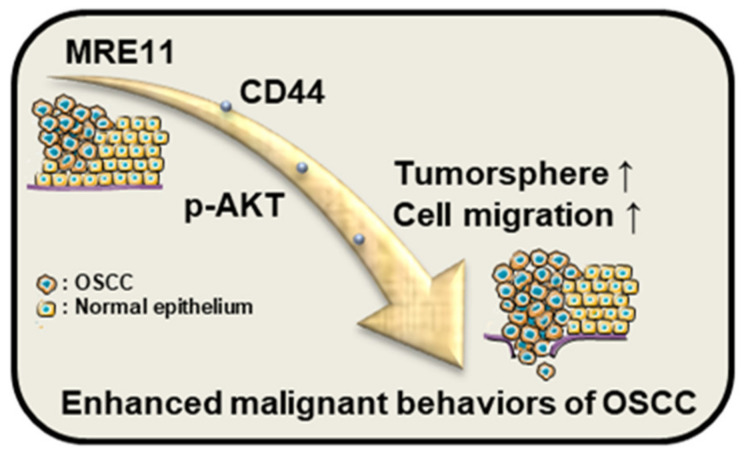
Schematic diagram for the MRE11-CD44-AKT pathway in OSCC cells. The results of the current study suggest that the CD44-associated AKT pathway may crucially mediate the MRE11-promoted malignant behaviors of OSCC cells, such as tumorsphere growth and cell migration ability.

The impact of cancer stemness on oral cancer management has been increasingly noted as the enhanced cancer stem cell-like properties, such as self-proliferation and migration ability, signify the potential of recurrence and metastasis [8,9,10], yet the molecular mechanisms with regard to these in OSCC cells remain to be elucidated. In this study, the role of CD44 under the influence of MRE11 in OSCC cells was investigated, implicating CD44 to be a crucial factor that mediates MRE11-promoted malignant behaviors of OSCC cells through AKT signaling. As summarized in Figure 5, the cancer-promoting effect via the route of MRE11-CD44-AKT may enhance tumorsphere growth and migration ability of OSCC cells. In addition, our clinical analysis and the results from xenograft metastatic mouse model further support these findings by unveiling a positive correlation between the expression of CD44 and AKT phosphorylation in the OSCC tumor tissues.

Our previous study showed that elevated MRE11 expression in OSCC patients was associated with adverse cancer progression, such as increased tumor stage and lymph node metastasis [24]. In addition, overexpression of MRE11 in OSCC cells exhibited enhanced cell proliferation and migration ability, whereas knockdown of MRE11 reduced these phenomena [24]. The current study further investigated the potential involvement of cancer stemness in these MRE11-promoting effects, leading to the findings of CD44 as a downstream effector of MRE11. While our current study provided evidence to reveal a novel pathway linking MRE11 and OSCC malignancy through enhancement of cancer stemness, there are also limitations in the interpretation of our data, and study areas to be further investigated. For example, the results of our in vivo investigation by the tail vein-injected metastatic model may be reinforced by an orthotopic xenograft model, where the spontaneous metastasis occurring from orthotopic tumor sites provides potential mimicry of the multi-step metastatic processes [38]. Furthermore, recent research from our group and others have noted the nuclease-independent functions of MRE11 in transcriptional activities [24,39], which are distinctive from the nuclease-dependent functions of MRE11 conventionally known for DNA damage repair. Future studies may also investigate into this to distinguish the involvement of nuclease activity in MRE11 that leads to upregulated CD44 expression in OSCC cells.

In conclusion, the newly identified pathway of MRE11-CD44-AKT unveiled in this study adds to the growing body of research on the significance of cancer stemness to OSCC malignancy, and these data may shed light on the development of novel therapeutic strategies, such as the potential via hyaluronic acid-based dual-targeting systems [40,41], to guide the drug delivery that simultaneously targets MRE11 or AKT in CD44-overexprssing OSCC.

## Figures and Tables

**Table 1:** Association of CD44 expression with clinicopathologic characteristics in OSCC patients.

	CD44		
Variable	Low	High	*p*-Value *
	*n* (%)	*n* (%)	
Age	41 (46.6)	47 (53.4)	–
years (mean ± SD)	52.0 ± 13.5	52.6 ± 13.2	0.830
Gender			
Female	4 (80.0)	1 (20.0)	0.280
Male	37 (44.6)	46 (55.4)	
Histologic grade			
I	37 (46.3)	43 (53.7)	0.866
II	4 (50.0)	4 (50.0)	
Tumor size			
T1 + T2	27 (43.5)	35 (56.5)	0.516
T3 + T4	14 (53.8)	12 (46.2)	
Lymph node metastasis			
No	35 (53.8)	30 (46.2)	0.040
Yes	6 (26.1)	17 (73.9)	
Pathologic stage			
I + II	23 (45.1)	28 (54.9)	0.910
III + IV	18 (48.6)	19 (51.4)	

* The *p* values were determined by Chi-square test for all variables except Age, which was determined by Student’s *t*-test. –, not applicable.

## Data Availability

The data presented in this study are available from the corresponding author upon reasonable request.

## Supplementary Materials

The following supporting information can be downloaded at: https://www.mdpi.com/article/10.3390/jpm12050841/s1, Figure S1. Oncomine database analysis for CD44 expression in normal and OSCC subjects; Figure S2. MRE11 regulates CD44 expression and tumorsphere formation in Ca9-22 OSCC cells; Figure S3. CD44 mediates MRE11-promoted AKT phosphorylation, tumorsphere formation, and cell migration in Ca9-22 OSCC cells.

## References

[1] Du M., Nair R., Jamieson L., Liu Z., Bi P. (2020). Incidence trends of lip, oral cavity, and pharyngeal cancers: Global burden of disease 1990–2017. J. Dent. Res..

[2] Rivera C. (2015). Essentials of oral cancer. Int. J. Clin. Exp. Pathol..

[3] Cheng Y., Li S., Gao L., Zhi K., Ren W. (2021). The molecular basis and therapeutic aspects of cisplatin resistance in oral squamous cell carcinoma. Front. Oncol..

[4] Vig N., Mackenzie I.C., Biddle A. (2015). Phenotypic plasticity and epithelial-to-mesenchymal transition in the behaviour and therapeutic response of oral squamous cell carcinoma. J. Oral Pathol. Med..

[5] Bai Y., Sha J., Okui T., Moriyama I., Ngo H.X., Tatsumi H., Kanno T. (2021). The epithelial-mesenchymal transition influences the resistance of oral squamous cell carcinoma to monoclonal antibodies via its effect on energy homeostasis and the tumor microenvironment. Cancers.

[6] Coletta R.D., Yeudall W.A., Salo T. (2020). Grand challenges in oral cancers. Front. Oral Health.

[7] Mughees M., Sengupta A., Khowal S., Wajid S. (2021). Mechanism of tumour microenvironment in the progression and development of oral cancer. Mol. Biol. Rep..

[8] Richard V., Sebastian P., Nair M.G., Nair S.N., Malieckal T.T., Santhosh Kumar T.R., Pillai M.R. (2013). Multiple drug resistant, tumorigenic stem-like cells in oral cancer. Cancer Lett..

[9] Sinha N., Mukhopadhyay S., Das D.N., Panda P.K., Bhutia S.K. (2013). Relevance of cancer initiating/stem cells in carcinogenesis and therapy resistance in oral cancer. Oral Oncol..

[10] Liu Y., Yang M., Luo J., Zhou H. (2020). Radiotherapy targeting cancer stem cells “awakens” them to induce tumour relapse and metastasis in oral cancer. Int. J. Oral Sci..

[11] Goodison S., Urquidi V., Tarin D. (1999). CD44 cell adhesion molecules. Mol. Pathol..

[12] Ponta H., Sherman L., Herrlich P.A. (2003). CD44: From adhesion molecules to signalling regulators. Nat. Rev. Mol. Cell Biol..

[13] Tahmasebi E., Alikhani M., Yazdanian A., Yazdanian M., Tebyanian H., Seifalian A. (2020). The current markers of cancer stem cell in oral cancers. Life Sci..

[14] Hassn Mesrati M., Syafruddin S.E., Mohtar M.A., Syahir A. (2021). CD44: A multifunctional mediator of cancer progression. Biomolecules.

[15] Morath I., Hartmann T.N., Orian-Rousseau V. (2016). CD44: More than a mere stem cell marker. Int. J. Biochem. Cell Biol..

[16] Wang S.J., Bourguignon L.Y. (2011). Role of hyaluronan-mediated CD44 signaling in head and neck squamous cell carcinoma progression and chemoresistance. Am. J. Pathol..

[17] Lee J.W., Lee H.Y. (2021). Targeting cancer stem cell markers or pathways: A potential therapeutic strategy for oral cancer treatment. Int. J. Stem Cells.

[18] Shahoumi L.A. (2021). Oral cancer stem cells: Therapeutic implications and challenges. Front. Oral Health.

[19] Hosoya N., Miyagawa K. (2014). Targeting DNA damage response in cancer therapy. Cancer Sci..

[20] Abad E., Graifer D., Lyakhovich A. (2020). DNA damage response and resistance of cancer stem cells. Cancer Lett..

[21] Williams R.S., Moncalian G., Williams J.S., Yamada Y., Limbo O., Shin D.S., Groocock L.M., Cahill D., Hitomi C., Guenther G. (2008). Mre11 dimers coordinate DNA end bridging and nuclease processing in double-strand-break repair. Cell.

[22] Wang Y.Y., Hung A.C., Lo S., Hsieh Y.C., Yuan S.F. (2021). MRE11 as a molecular signature and therapeutic target for cancer treatment with radiotherapy. Cancer Lett..

[23] Situ Y., Chung L., Lee C.S., Ho V. (2019). MRN (MRE11-RAD50-NBS1) complex in human cancer and prognostic implications in colorectal cancer. Int. J. Mol. Sci..

[24] Wang Y.Y., Chen Y.K., Lo S., Chi T.C., Chen Y.H., Hu S.C., Chen Y.W., Jiang S.S., Tsai F.Y., Liu W. (2021). MRE11 promotes oral cancer progression through RUNX2/CXCR4/AKT/FOXA2 signaling in a nuclease-independent manner. Oncogene.

[25] Johnson S., Chen H., Lo P.K. (2013). In vitro tumorsphere formation assays. Bio-Protocol.

[26] Huang J.Y., Wang Y.Y., Lo S., Tseng L.M., Chen D.R., Wu Y.C., Hou M.F., Yuan S.F. (2020). Visfatin mediates malignant behaviors through adipose-derived stem cells intermediary in breast cancer. Cancers.

[27] Wang Y.Y., Chen H.D., Lo S., Chen Y.K., Huang Y.C., Hu S.C., Hsieh Y.C., Hung A.C., Hou M.F., Yuan S.F. (2020). Visfatin enhances breast cancer progression through CXCL1 induction in tumor-associated macrophages. Cancers.

[28] Wang Y.Y., Vadhan A., Chen P.H., Lee Y.L., Chao C.Y., Cheng K.H., Chang Y.C., Hu S.C., Yuan S.F. (2021). CD44 promotes lung cancer cell metastasis through ERK-ZEB1 signaling. Cancers.

[29] Rhodes D.R., Yu J., Shanker K., Deshpande N., Varambally R., Ghosh D., Barrette T., Pandey A., Chinnaiyan A.M. (2004). ONCOMINE: A cancer microarray database and integrated data-mining platform. Neoplasia.

[30] Emich H., Chapireau D., Hutchison I., Mackenzie I. (2015). The potential of CD44 as a diagnostic and prognostic tool in oral cancer. J. Oral Pathol. Med..

[31] Xu H., Niu M., Yuan X., Wu K., Liu A. (2020). CD44 as a tumor biomarker and therapeutic target. Exp. Hematol. Oncol..

[32] Weiswald L.B., Bellet D., Dangles-Marie V. (2015). Spherical cancer models in tumor biology. Neoplasia.

[33] Bates R.C., Edwards N.S., Burns G.F., Fisher D.E. (2001). A CD44 survival pathway triggers chemoresistance via lyn kinase and phosphoinositide 3-kinase/Akt in colon carcinoma cells. Cancer Res..

[34] Yu S., Cai X., Wu C., Wu L., Wang Y., Liu Y., Yu Z., Qin S., Ma F., Thiery J.P. (2015). Adhesion glycoprotein CD44 functions as an upstream regulator of a network connecting ERK, AKT and Hippo-YAP pathways in cancer progression. Oncotarget.

[35] Gomez K.E., Wu F., Keysar S.B., Morton J.J., Miller B., Chimed T.S., Le P.N., Nieto C., Chowdhury F.N., Tyagi A. (2020). Cancer cell CD44 mediates macrophage/monocyte-driven regulation of head and neck cancer stem cells. Cancer Res..

[36] Brabletz T., Jung A., Spaderna S., Hlubek F., Kirchner T. (2005). Migrating cancer stem cells—An integrated concept of malignant tumour progression. Nat. Rev. Cancer.

[37] Ingangi V., Minopoli M., Ragone C., Motti M.L., Carriero M.V. (2019). Role of microenvironment on the fate of disseminating cancer stem cells. Front. Oncol..

[38] Nakayama J., Ito E., Fujimoto J., Watanabe S., Semba K. (2017). Comparative analysis of gene regulatory networks of highly metastatic breast cancer cells established by orthotopic transplantation and intra-circulation injection. Int. J. Oncol..

[39] Sharma S., Anand R., Zhang X., Francia S., Michelini F., Galbiati A., Williams H., Ronato D.A., Masson J.Y., Rothenberg E. (2021). MRE11-RAD50-NBS1 complex is sufficient to promote transcription by RNA polymerase II at double-strand breaks by melting DNA ends. Cell Rep..

[40] Dosio F., Arpicco S., Stella B., Fattal E. (2016). Hyaluronic acid for anticancer drug and nucleic acid delivery. Adv. Drug Deliv. Rev..

[41] Duan H., Liu Y., Gao Z., Huang W. (2021). Recent advances in drug delivery systems for targeting cancer stem cells. Acta Pharm. Sin. B.

