# Supplementary material for: CD44 Mediates Oral Squamous Cell Carcinoma-Promoting Activity of MRE11 via AKT Signaling
Source: J Pers Med. 2022 May 21;12(5):841. doi: 10.3390/jpm12050841 (PMC9144890; doi:10.3390/jpm12050841)
Supplement: Supplementary file 1 [file jpm-12-00841-s001.zip › jpm-1698596-supplementary.pdf]

Supplementary Materials

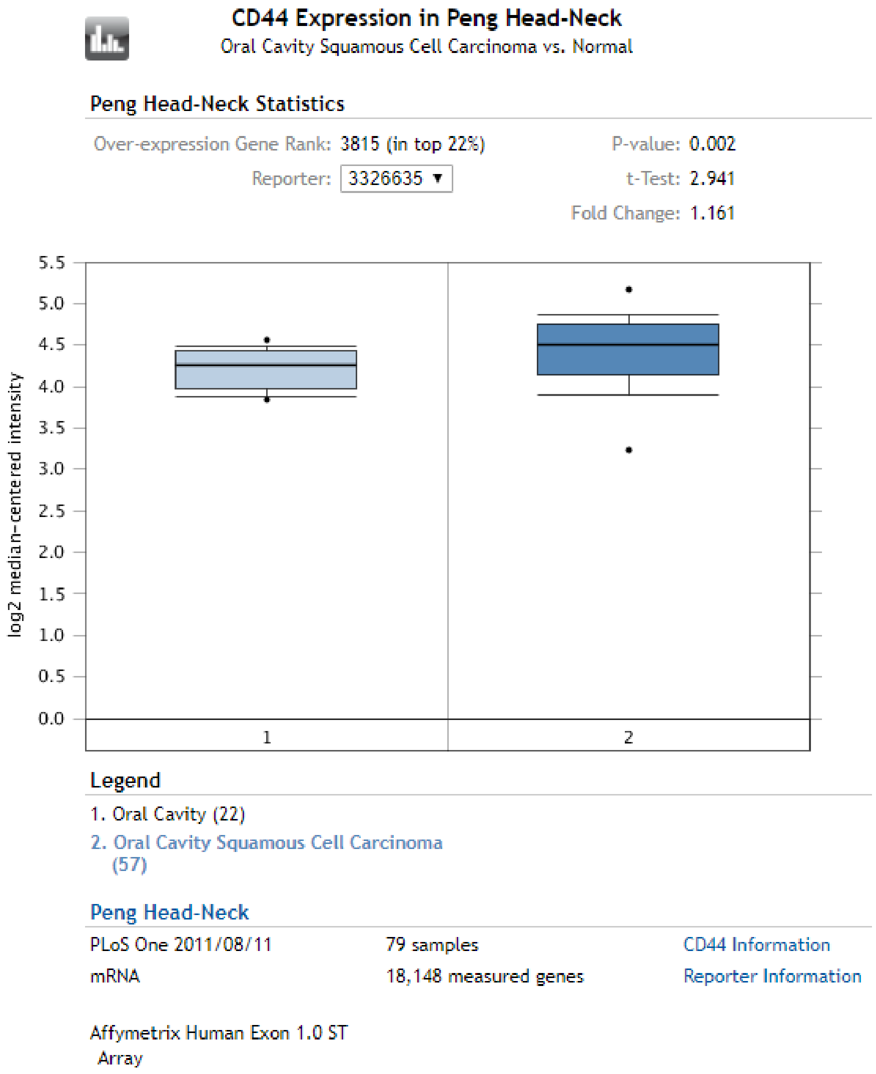

Supplementary Figure S1. Oncomine database analysis for CD44 expression in normal and OSCC subjects. CD44 mRNA expression in normal oral tissues and OSCC tumor tissues was analyzed and retrieved using the Oncomine database. Data were presented as boxplots for normal oral tissues (n = 22) and OSCC tumor tissues (n = 57). Statistical difference was determined by Student's t-test (p = 0.002).

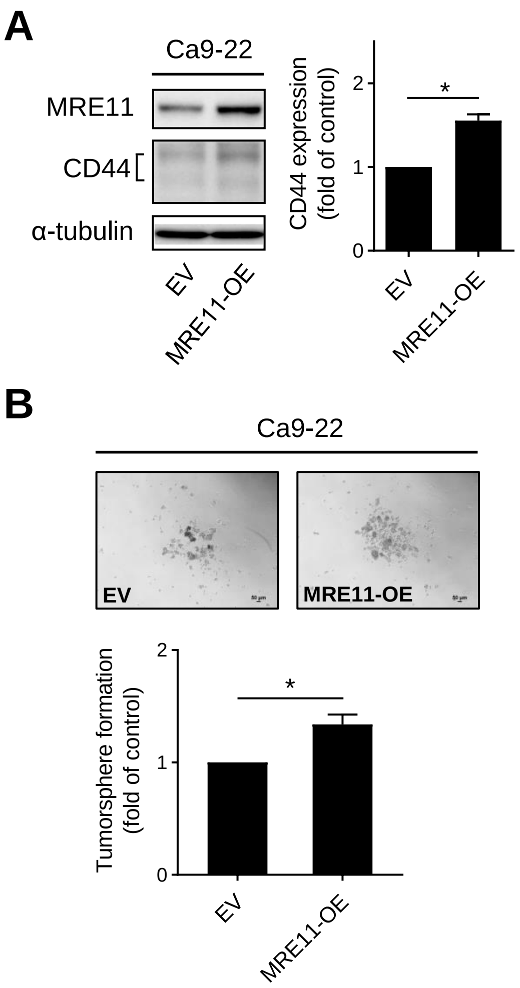

Supplementary Figure S2. MRE11 regulates CD44 expression and tumorsphere formation in Ca9-22 OSCC cells. (**A**) Total protein lysates from Ca9-22 cells carrying overexpression vector of MRE11 (MRE11-OE) or empty vector (EV) were collected and analyzed by Western blot. (**B**) Ca9-22 cells carrying overexpression vector of MRE11 (MRE11-OE) or empty vector (EV) were collected and re-plated in ultra-low attachment microplates for tumorsphere formation assay. Quantitation of tumorspheres was carried out for those with diameter > 50 µm. Data were obtained from three independent experiments and presented as mean ± SEM. Statistical difference was determined by Student's t-test. $^{*}p < 0.05$.

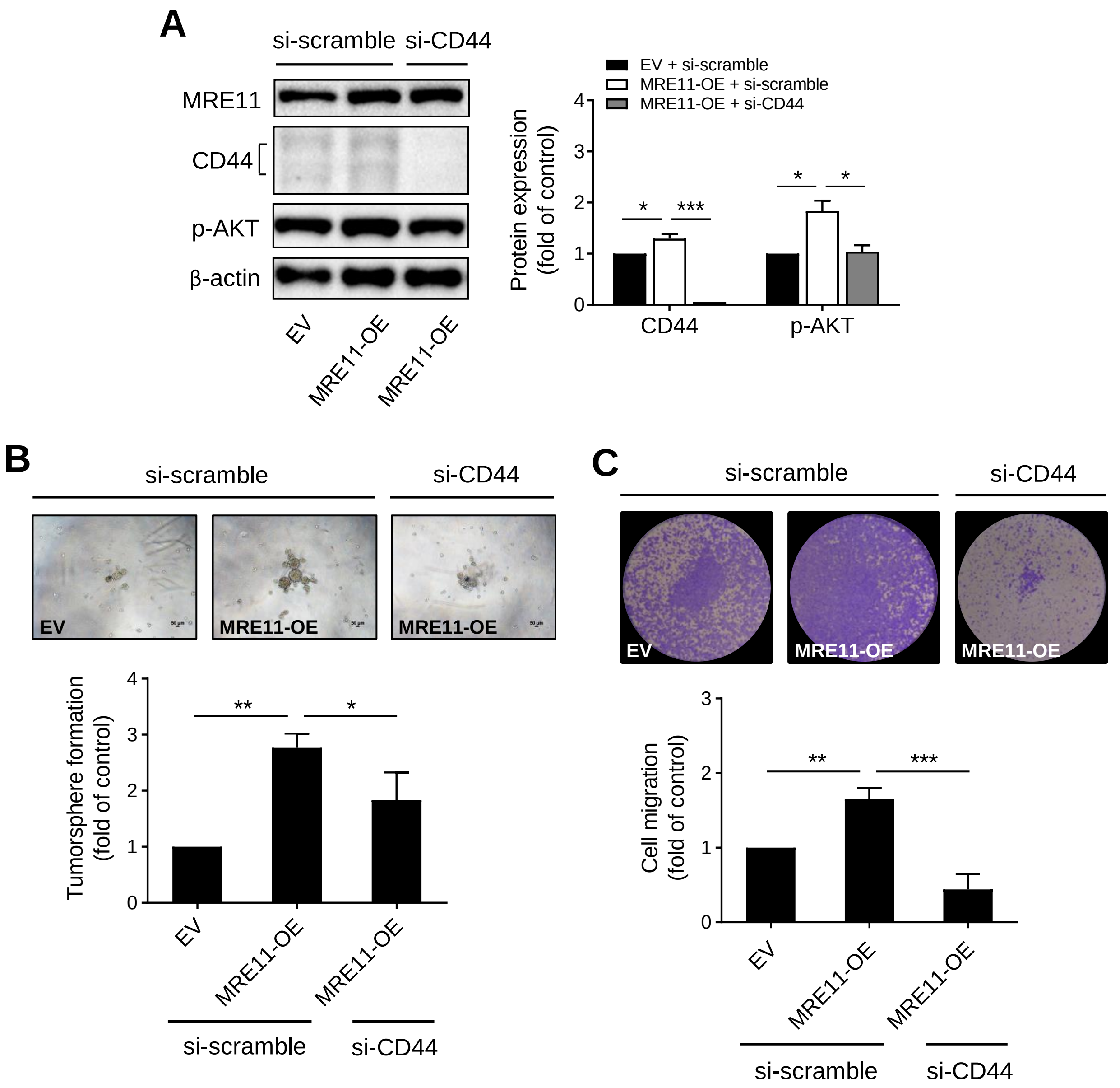

Supplementary Figure S3. CD44 mediates MRE11-promoted AKT phosphorylation, tumorsphere formation, and cell migration in Ca9-22 OSCC cells. (**A**) Total protein lysates from Ca9-22 cells carrying (i) empty vector (EV) and siRNA containing a scramble sequence (si-scramble), (ii) overexpression vector of MRE11 (MRE11-OE) and siRNA containing a scramble sequence (si-scramble), or (iii) overexpression vector of MRE11 (MRE11-OE) and siRNA targeting CD44 (si-CD44), were collected and analyzed by Western blot. (**B, C**) Ca9-22 cells carrying (i) empty vector (EV) and siRNA containing a scramble sequence (si-scramble), (ii) overexpression vector of MRE11 (MRE11-OE) and siRNA containing a scramble sequence (si-scramble), or (iii) overexpression vector of MRE11 (MRE11-OE) and siRNA targeting CD44 (si-CD44), were collected and re-plated in ultra-low attachment microplates for tumorsphere formation assay in (**B**), or re-plated in the insert of transwell plates for transwell cell migration assay in (**C**). Quantitation of tumorspheres was carried out for those with diameter > 50 µm. Data were obtained from three independent experiments and presented as mean ± SEM. Statistical difference was determined by Student's t-test. $^{*}p < 0.05$, $^{**}p < 0.01$, $^{***}p < 0.001$.
